# Large-cell neuroendocrine carcinoma of the common bile duct: a case report and a review of literature

**DOI:** 10.1186/s40792-016-0269-8

**Published:** 2016-11-26

**Authors:** Makoto Murakami, Kanji Katayama, Shigeru Kato, Daisuke Fujimoto, Mitsuhiro Morikawa, Kenji Koneri, Yasuo Hirono, Takanori Goi

**Affiliations:** First Department of Surgery, School of Medicine, University of Fukui, 23 Shimoaiduki, Matsuoka, Eiheiji-cho, Fukui, 910-1193 Japan

**Keywords:** Neuroendocrine tumor, Large-cell neuroendocrine carcinoma, Common bile duct

## Abstract

**Background:**

Large-cell neuroendocrine carcinoma (LCNEC) of the bile duct is extremely rare and is a high-grade type of neuroendocrine tumor with an aggressive clinical course. Here, we report a case of LCNEC of the extrahepatic bile duct.

**Case presentation:**

An 80-year-old man presented with severe jaundice. Endoscopic retrograde cholangiography and enhanced computed tomography revealed complete obstruction of the common bile duct (CBD) by a dense tumor measuring 1.5 cm in diameter. Although there were no malignant cells in the biliary brush cytology, we suspected a cholangiocarcinoma and performed extrahepatic bile duct resection. Histologically, the LCNEC occupied most of the places deeper than the stratum submucosum and an adenocarcinoma component, approximately 15%, was present in the mucosa. There were no transitional areas between the two components. Immunohistochemically, the LCNEC cells were reactive for CD56 and synaptophysin and had a high MIB-1 index (72%). The patient died of multiple liver, lung, and peritoneal metastases 3 months after surgery.

**Conclusions:**

LCNEC of the CBD is particularly rare and has a very poor prognosis. Only five cases have been reported in the literature; therefore, there is no established effective therapy, including surgery, for LCNEC of the CBD at present. An accumulation of additional cases and further studies of multimodal treatment are required in the future to improve the prognosis.

## Background

Primary neuroendocrine carcinoma (NEC) of the common bile duct is rare, and only 27 cases have been reported in the literature so far. One type of NEC, large-cell neuroendocrine carcinoma (LCNEC), is particularly rare, and only five cases have been previously reported. Here, we report a case of LCNEC of the extrahepatic bile duct and a review of the literature.

## Case presentation

An 80-year-old man presented to our hospital with anorexia and jaundice that had been present for several weeks. The patient had neither abdominal tenderness nor a palpable mass in the right upper quadrant of the abdomen. He underwent endoscopic submucosal dissection for multiple early gastric cancers 1 year ago, and all of the lesions were resected radically. He had severe chronic obstructive pulmonary disease because of long-term smoking. The patient’s main occupation was in agriculture, and he had never worked in the printing or staining industries. The patient had no family history of cancer.

On the day of admission, his complete blood count and serum biochemical parameters were as follows: white blood cells, 10.5 × 10^3^/μL (normal range 3.3–9.0 × 10^3^/μL); red blood cells, 28.5 × 10^6^/μL (4.3–5.7 × 10^6^/μL); hemoglobin, 9.2 g/dL (13.5–17.5 g/dL); total protein, 5.0 g/dL (6.7–8.3 g/dL); albumin, 1.5 g/dL (4.0–5.0 g/dL); total bilirubin, 26.0 mg/dL (0.3–1.1 mg/dL); direct bilirubin, 17.0 mg/dL (0.1–0.5 mg/dL); aspartate aminotransaminase, 91 IU/L (normal 10–40 IU/L); alanine aminophosphatase, 90 IU/L (5–45 IU/L); aspartate transferase, 1560 IU/L (100–325 IU/L); and gamma-glutamyltranspeptidase, 278 IU/L (7–80 IU/L). His carbohydrate antigen 19-9 (CA19-9) was 40,635 U/mL (0–37 U/mL), and carcinoembryonic antigen (CEA) was 10.4 ng/mL (0–5.0 ng/mL). Alpha fetoprotein levels were within normal limits.

**Fig. 1:**
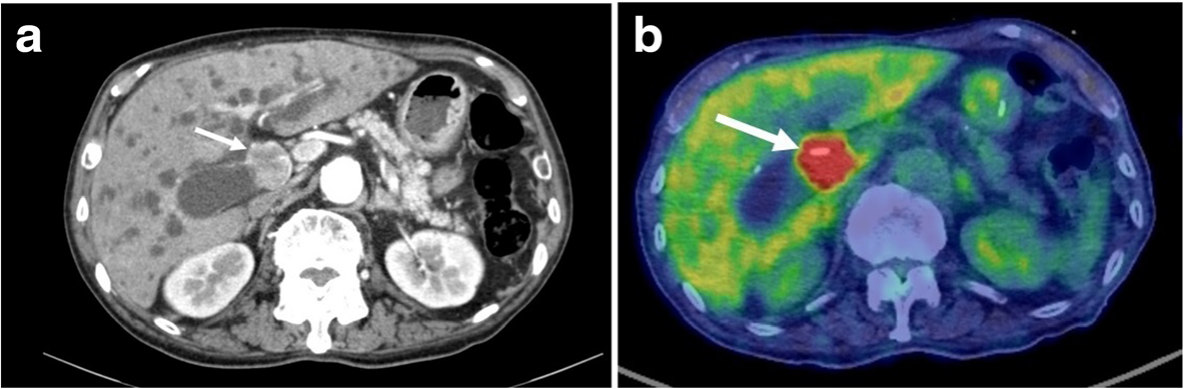
CT (early phase) and FDG-PET findings. **a** CT showed an enhanced mass that was approximately 2.5 cm in size located in the mid-CBD (*arrow*). **b** FDG-PET showed a high accumulation of FDG with a maximum standardized uptake value (SUVmax) of 20.7 in the CBD tumor (*arrow*)

An abdominal computed tomography (CT) scan revealed an enhanced mass that was approximately 2.5 cm in size located in the mid common bile duct (CBD) and an enlarged regional node in the hepatoduodenal ligament (Fig. 1a). ^18^F-fluorodeoxyglucose positron emission tomography (FDG-PET) revealed high accumulation of FDG with a maximum standardized uptake value (SUVmax) of 20.7 by the CBD tumor (Fig. 1b).

**Fig. 2:**
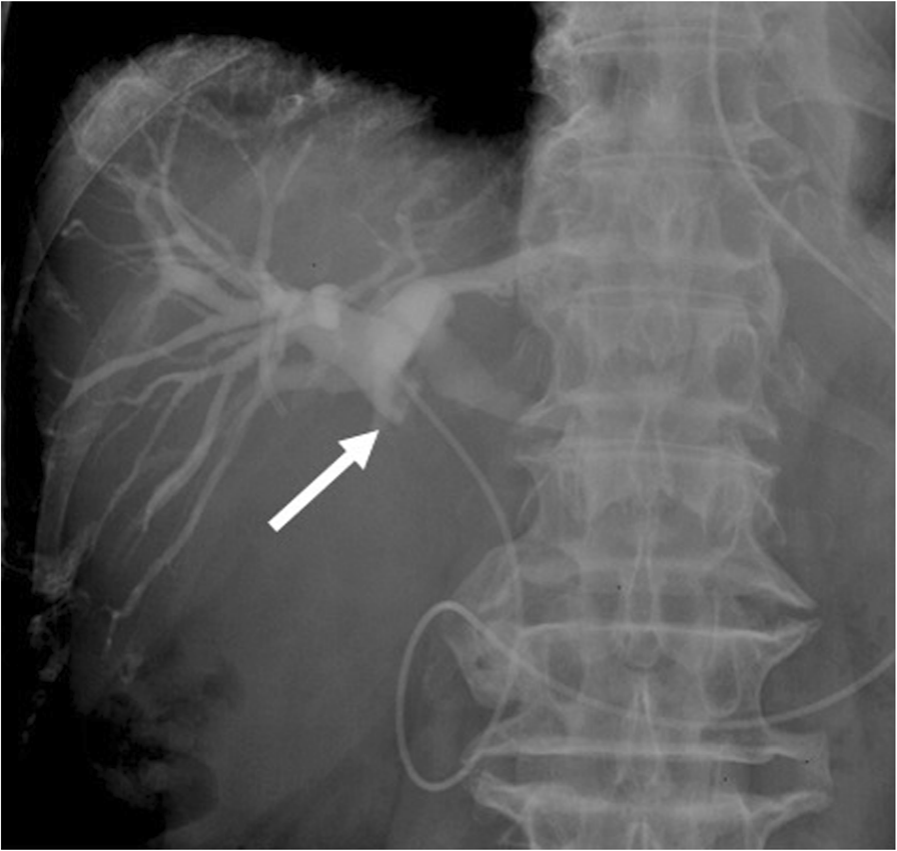
Cholangiopancreatography findings. Cholangiography revealed severe intrahepatic bile duct dilatation and a filling defect in the mid-CBD (*arrow*). The tumor involved with the cystic duct

Endoscopic retrograde cholangiopancreatography (ERCP) demonstrated severe intrahepatic bile duct dilatation and a filling defect in the mid-CBD. The tumor was involved with the cystic duct. After the ERCP, endoscopic placement of a nasobiliary biliary drainage catheter was performed (Fig. 2). The bile and brush cytology performed at the same time revealed a few degenerative atypical cells (class 3).

The patient underwent surgery with the presumed diagnosis of bile duct cancer. The operative method was extrahepatic bile duct resection with lymph node dissection and reconstruction with a Roux en Y hepaticojejunostomy because of his advanced age and severe pulmonary emphysema. The 18 regional lymph nodes were resected. The right hepatic artery was fixed to the tumor, and we performed a complicated resection. There was evidence of local cancer invasion, but no signs of distant metastasis or organ invasion were noted. The resection margins of the proximal and distal bile duct frozen biopsy were tumor free. Peritoneal lavage cytology during the operation was negative.

**Fig. 3:**
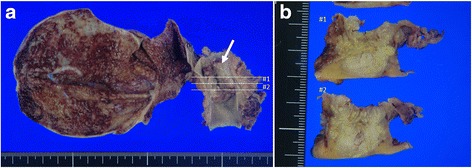
Formalin-fixed surgical specimen. **a** The surgical specimen showed a tumor measuring 2.4 × 1.9 cm located in the mid-CBD (*arrow*). **b** The sectioned surface (at #1 and #2 of Fig. 1) of the resected specimen showed tumor invasion beyond the bile duct serosa

Macroscopically, the surgical specimen showed a light gray invasive nodular tumor measuring 2.4 × 1.9 cm located in the mid-CBD (Fig. 3a). The sectioned surface of the resected specimen showed tumor invasion beyond the bile duct serosa (Fig. 3b). There were no gallstones in either the gallbladder or bile ducts.

**Fig. 4:**
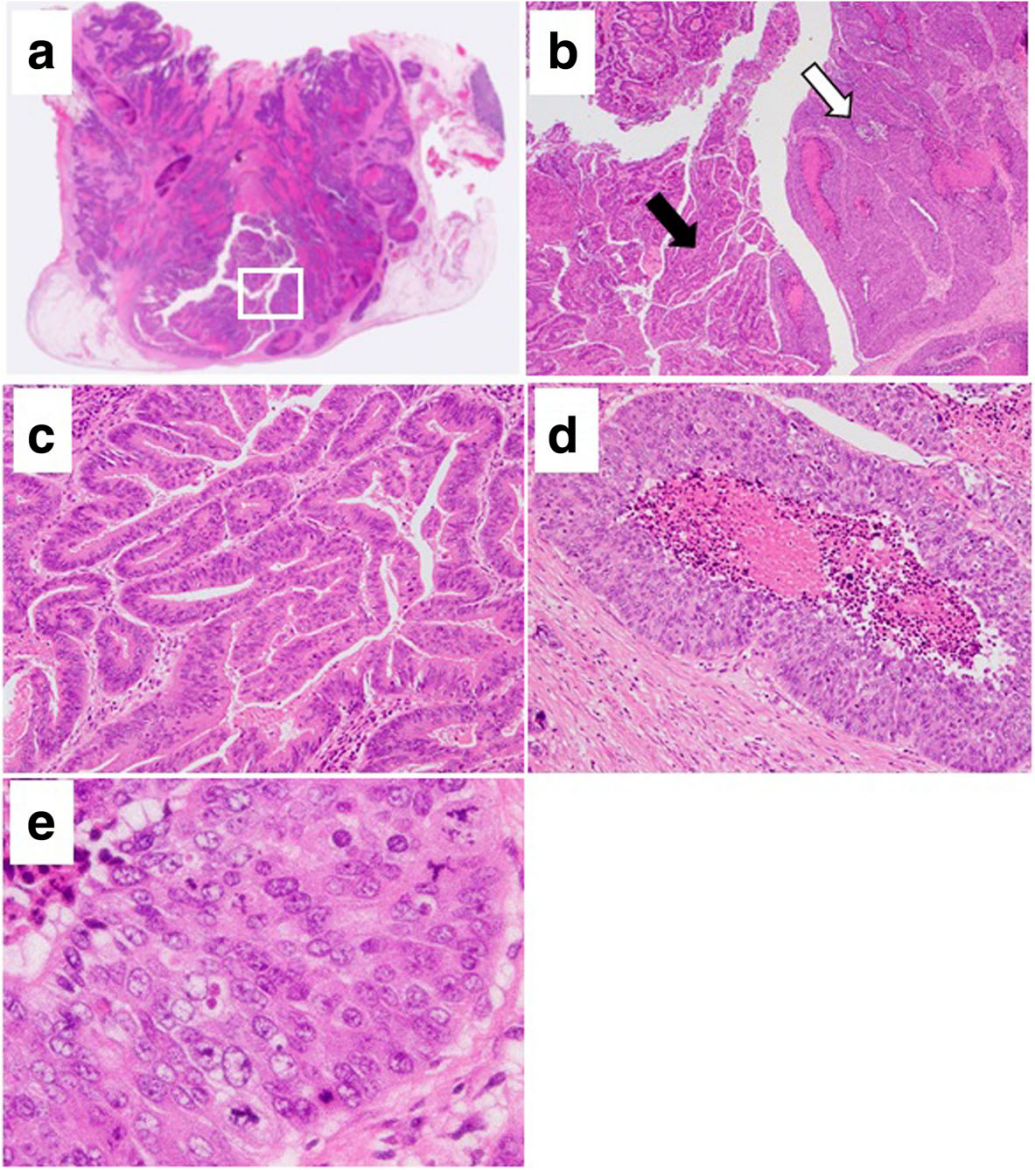
Pathologic examination of the resected specimen stained with hematoxylin and eosin. **a** Loupe image of the tumor revealed invasion throughout the entire CBD wall with serosal penetration. **b** Magnification of the part in the *square* in **a**. The tumor was structured with two components (*black* and *white arrows*). There was no transitional area between the two components. **c** Intermediate-magnification image of the part marked with a *black arrow* showed moderate differentiated adenocarcinoma. **d** The intermediate-magnification image of the part marked with a *white arrow* shows LCNEC, which made up approximately 90% of the tumor. The tumor was solid and cellular with necrosis inside. The tumor cells were joined together, and the cytoplasm was relatively large. **e** A high magnification of **d** shows that each nucleus variant was large and the heteromorphic nuclei division image was obvious

On histopathological examination of the resected specimen stained with hematoxylin and eosin, the tumor was structured with two components (Fig. 4a). There was no transitional area between the two components. High cellularity components made up approximately 90% of the tumor and exhibited invasion throughout the entire CBD wall with serosal penetration (Fig. 4b). The other component was tubular adenocarcinoma, and it occupied a small area of the tumor in the superficial mucosal portion (Fig. 4c). In the LCNEC area, the tumor was solid and cellular with necrosis inside. There was no differentiation into duct structures. The tumor cells were joined together, and the cells, nucleus, and cytoplasm were relatively large (Fig. 4d). A high-power image of d shows that the tumor cells were large idioblasts and the nucleolus was clear. Each nucleus variant was strong, and the heteromorphic nuclei division image was obvious (Fig. 4e).

**Fig. 5:**
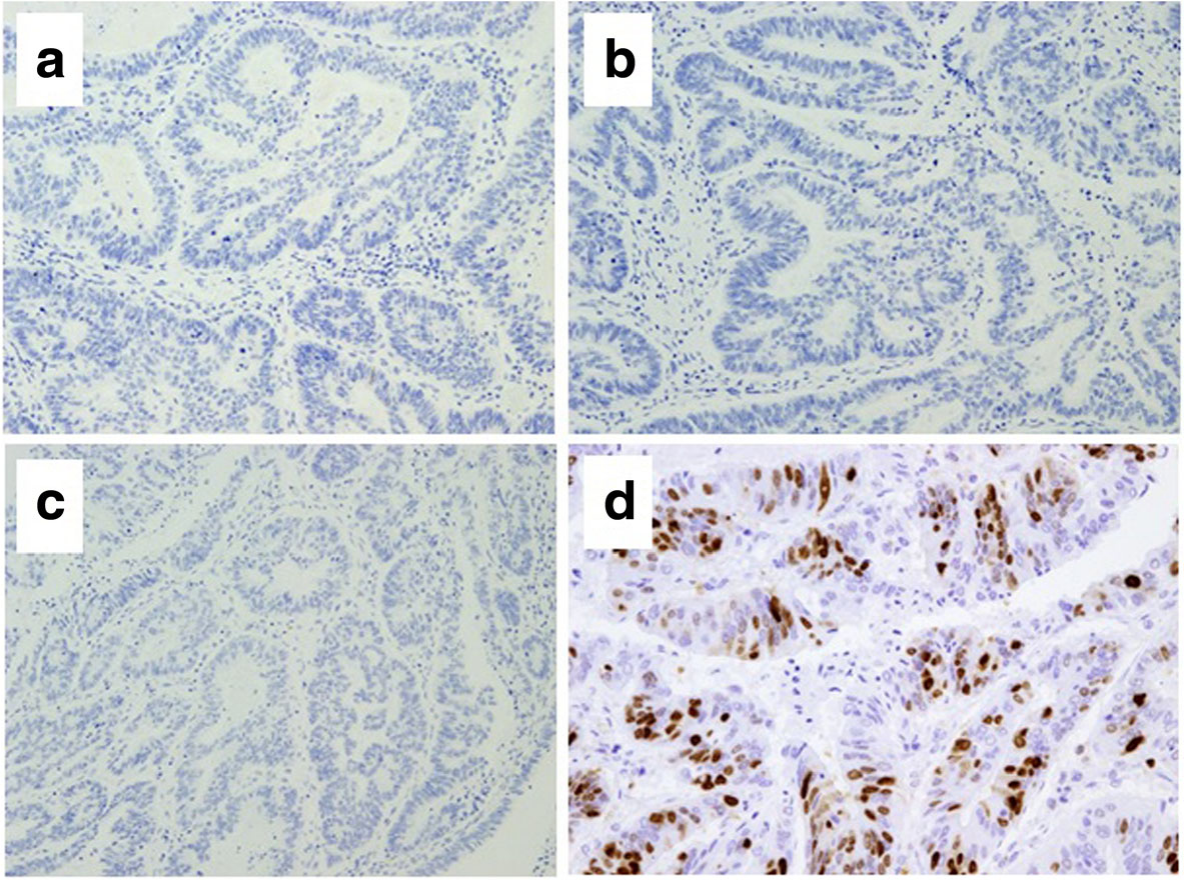
Immunohistochemical findings in the component of LCNEC. **a** Immunostaining for synaptophysin was partially positive. **b** Immunostaining for chromogranin A was negative. **c** Immunostaining for CD56 was strongly positive in most of the LCNEC cells. **d** Immunostaining for Ki-67 was strongly positive in 72% of the LCNEC cells

**Fig. 6:**
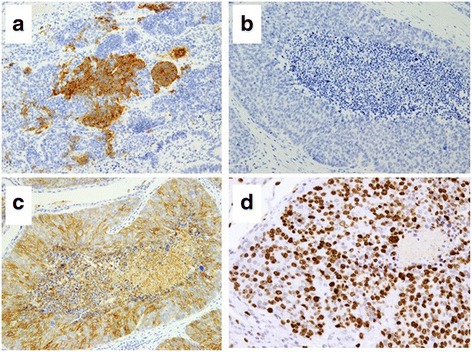
Immunohistochemical findings in the component of adenocarcinoma. There were no immunopositive cells in the adenocarcinoma component. **a** Immunostaining for synaptophysin was negative. **b** Immunostaining for chromogranin A was negative. **c** Immunostaining for CD56 was negative. **d** Immunostaining for Ki-67 showed diffused positivity in 27% of the adenocarcinoma cells

Immunohistochemical findings in the LCNEC component indicated that the tumor cells were immunopositive for neuroendocrine markers, including synaptophysin and CD56, but were negative for chromogranin A and neurospecific enolase (NSE) (Fig. 5a–c). Immunostaining for Ki-67 showed a strong positive of 72% (Fig. 5d). Immunohistochemical findings in the adenocarcinoma component indicated that the tumor cells were not immunopositive for neuroendocrine markers (Fig. 6a–c). There were no transitional areas between the components. Staining for Ki-67 showed mild positive at 27% (Fig. 6d). Metastases from the LCNEC were noted in two of the 18 lymph nodes. The metastatic lymph nodes were in contact with the tumor.

No postoperative complications occurred, and the patient was discharged. His CEA and CA19-9 levels normalized after the operation.

The patient had peritoneal metastases develop during the early postoperative period, and a postoperative CT only 2.5 months later showed a lung metastasis and multiple liver metastases occupying half of the liver. The patient died 3 months after surgery.

### Discussion

In the World Health Organization classification, neuroendocrine neoplasms are classified into five general categories, including neuroendocrine tumor (NET), NEC, mixed adenoneuroendocrine carcinoma (MANEC), goblet cell carcinoid, and tubular carcinoid. In addition, NECs are classified as either LCNEC or small-cell neuroendocrine carcinoma (SCNEC) [1]. When each component is more than 30% of the tumor, it is defined as MANEC.

The bile ducts are one of the rarest primary organs for NET, accounting for only 0.2 to 2.0% of all such tumors [2]. NEC arising in the extrahepatic bile duct includes pure NEC, MANEC, and NEC with adenocarcinoma, but only 27 cases have been described previously in the literature [3–29] (Table 1). Of these, 19 cases were pure NEC and eight cases were composite glandular–endocrine cell carcinoma of the extrahepatic bile ducts. Most of these cases (82%) were SCNEC, and LCNEC was extremely rare, only five cases. Sato et al. first reported LCNEC with adenocarcinoma in the CBD in 2006 [16], and thus, our case becomes the sixth report of LCNEC arising in the extrahepatic bile duct.

**Table 1:** Reported cases of neuroendocrine carcinoma of the extrahepatic bile duct. Review of the literature

No.	Author	Age	Sex	Histology	Location	Size	Treatment	Prognosis
1	Sabanathan [3]	67	M	SCNEC	Bm	5 cm	Palliative bypass and chemo.	6 m, alive
2	Van der Wal [4]	55	M	SCNEC + Adenoca.	Bm	4 cm	Resection	N.A.
3	Nishihara [5]	64	M	SCNEC + Adenoca.	Bh-Bs	1.9 cm	Resection	8 m, alive
4	Yamamoto [6]	71	F	SCNEC + Adenoca.	Bh	6 cm	Resection	8 m, dead
5	Kim [7]	64	M	SCNEC + Adenoca.	Bm	3 cm	Resection	1 m, alive
6	Miyashita [8]	85	F	SCNEC	Bi	3 cm	Palliative bypass	5 m, dead
7	Edakuni [9]	82	F	SCNEC + Adenoca.	Bm	6 cm	Resection	45 m, alive
8	Kuraoka [10]	75	M	SCNEC	Bi	4.5 cm	Resection	5 m, alive
9	Hazama [11]	60	M	SCNEC	CBD	0.3 cm	NAC and resection	12 m, dead
10	Arakura [12]	70	F	SCNEC	Bm	3 cm	Resection and chemo.	14 m, dead
11	Park [13]	60	F	SCNEC	Bs-Bm	3 cm	Resection	5 m, dead
12	Thomas [14]	54	M	SCNEC	Bh-Bm	N.A.	Resection	6 m, alive
13	Kaiho [15]	66	F	SCNEC + Adenoca.	Bm	3.5 cm	Resection and chemo.	8 m, dead
14	Sato [16]	68	M	LCNEC + Adenoca.	Bi	2 cm	Resection and chemo.	3 m, dead
15	Viana Miguel [17]	76	M	SCNEC	Bm	N.A.	Resection, chemo., and irradiation	5 m, alive
16	Jeon [18]	65	M	SCNEC	Bs-Bm	2 cm	Resection and chemo.	12 m, dead
17	Nakai [19]	32	M	SCNEC	CBD	N.A.	N.A.	N.A. (autopsy)
18	Arakura [20]	75	M	SCNEC	Bh-Bs	6.5 cm	Chemo. and irradiation	10 m, dead
19	Hosonuma [21]	69	F	SCNEC	Bs-Bm	3 cm	Biliary drainage	2 m, alive
20	Okamura [22]	62	M	SCNEC	Bm	3 cm	NAC, resection, and irradiation	20 m, dead
21	Yamaguchi [23]	77	F	NEC	Bi	N.A.	Resection and chemo.	27 m, alive
22	Demoreuil [24]	73	M	LCNEC + Adenoca.	Bh-Bs	3 cm	Resection and chemo.	12 m, dead
23	Cho [25]	59	F	SCNEC	Bm	3 cm	Resection	6 m, dead
24	Sasatomi [26]	76	M	LCNEC	Bh-Bs	5 cm	Resection	21 days, dead
25	Ninomiya [27]	75	F	LCNEC	Bm	3 cm	Resection	14 m, alive
26	Park [28]	75	F	LCNEC	Bm	2.7 cm	Resection and chemo.	12 m, dead
27	Kihara [29]	70	F	SCNEC	Bh	5 cm	Resection and chemo.	10 m, alive
28	Current report	79	M	LCNEC + Adenoca.	Pm	2.9 cm	Resection	3 m, dead

*NEC* neuroendocrine carcinoma, *NAC* neoadjuvant chemotherapy, *Adenoca.* adenocarcinoma, *CBD* common bile duct, *Bh* hilar bile duct, *Bs* superior portion of common bile duct, *Bm* mid-portion of bile duct, *Bi* inferior portion of bile duct, *chemo*. chemotherapy, *N.A.* not available

Sasatomi (2013), Ninomiya (2013), and Park (2014) reported cases of pure LCNEC in the CBD [26–28]. From what can be analyzed from the literature, the mean tumor diameter was 3.5 cm (range 0.3–6.5 cm), median survival time was 12.0 months (range 0.7–45 m), and the 1-year survival rate was 32.6%. In 84% of cases, radical resection was performed.

The pathological fact that normal bile duct mucosa does not have neuroendocrine cells was cited as one of the reasons why a primary NEC of the CBD is extremely rare. Harada et al. examined 274 cases of biliary cancer and reported that MANET was found in 4% of hepatic hilar cholangiocarcinomas with hepatolithiasis, 10% of gallbladder cancers, and 4% of extrahepatic cholangiocarcinomas [30]. The authors of this report expressed the opinion that normal adenocarcinoma developed during a process of growth and dedifferentiation to endocrine cells. Albores et al. reported that neuroendocrine cells could be detected at sites of intestinal metaplasia induced by chronic inflammation due to cholelithiasis and congenital anomalies, which might be the initial step in the development of neuroendocrine tumors of the CBD [31]. The process suggested this report was one reason why pure NET and NEC developed. Although pure NET cases without dysplastic intestinal-type epithelium exist, they seem to follow a different developmental process.

LCNEC of the CBD is a poorly differentiated and rare tumor that exhibits high-grade NET with aggressive behavior and has a strong tendency to develop early lymph node and distant metastases. The survival duration of previously reported cases of LCNEC of the CBD ranged from only 21 days to 12 months after surgery (Table 1). In our case, the recurrence was noted 2.5 months after surgery, and the patient died 3 weeks after the recurrence. The prognosis of LCNEC is extremely poor in comparison with adenocarcinoma of the same clinical stage, even if we can resect the tumor radically.

In this case, we chose to perform extrahepatic bile duct resection, which is not commonly used. In cases of extrahepatic bile duct cancer with obstruction of the cystic duct, we usually perform subtotal stomach-preserving pancreatoduodenectomy because of metastasis of lymph nodes around the head of the pancreas and direct invasion to the pancreatic parenchyma. Because the patient was 80 years old and had progressive dementia and severe pulmonary emphysema, once the resection margin of the distal CBD frozen biopsy was tumor free, with the consent of his family, we decided to defer pancreatoduodenectomy. We do not believe that this reduction surgery caused early postoperative liver metastasis.

Adjuvant chemotherapy in three cases of LCNEC of the CBD was reported previously [16, 24, 27], and these patients survived 3 to 12 months. Yamaguchi et al. reported that adjuvant chemotherapy with gemcitabine could not suppress the recurrence, but hepatic artery infusion with CPT-11 (40 mg/kg body weight) and CDDP (20 mg/kg body weight) every 2 weeks remarkably decreased tumor markers and the size of both lymph nodes and liver tumors [23].

At present, it is controversial whether a combination of chemotherapy and radiotherapy is more effective than resection alone [17, 22]. In this case, we could not provide adjuvant chemotherapy because of the general poor condition of the patient. Because peritoneal, lung, and liver metastases developed during the early postoperative period, it was hard to attempt any adjuvant chemotherapy in LCNEC. If a diagnosis of LCNEC of the CBD was possible preoperatively, we could consider neoadjuvant multimodal treatments before resection to improve the prognosis [11, 21, 32]. However, it was difficult to diagnose NEC of the CBD preoperatively, because there is no difference between adenocarcinoma and NEC in symptoms, blood tests, and imaging studies. In most cases, the definitive diagnosis was established by histopathological and immunohistochemical analysis of the surgical specimen. Only three cases were diagnosed preoperatively by percutaneous transhepatic cholangioscopy with biopsy [11], ERCP with brushing [14], and endoscopic ultrasonography-guided fine-needle aspiration biopsy [12]. In our case, we were not able to detect malignant cells by brush cytology of the bile duct. The submucosal location of NEC causes a large number of false-negative results on brush biopsy, making it difficult to achieve a correct preoperative diagnosis.

In our case, CA19-9 was elevated to a high level (40,635 U/mL), but it was thought that the abnormal value was caused by chronic cholestasis and cholangitis. The patient’s postoperative values of CA19-9 reduced to a normal level immediately and did not correlate with the cancer recurrence.

Cho et al. suggested that NEC of the CBD should be considered in differential diagnosis of causes of obstructive jaundice and hemobilia [25]. We examined the intraductal ultrasonography of the CBD in our case and detected a large quantity of clots in the lower bile duct. It was thought that this appearance was due to LCNEC, which was often associated with necrosis. The Ki-67 index in the LCNEC component was higher than in the adenocarcinoma component (72 vs. 27%), and probably reflecting this difference, the SUVmax of the LCNEC was high (20.7) on the FDG-PET. When the SUVmax reaches an abnormally high level, it seems reasonable to suspect a different type of carcinoma.

## Conclusions

In summary, we report a case of LCNEC of the CBD. This disease is extremely rare and has an aggressive malignant potential, including invasiveness and metastasis. There are no effective treatments, including resection. Accumulation of more cases and further studies of multimodal treatment are required to improve the prognosis of patients with this disease.

## Abbreviations

CA19-9: Carbohydrate antigen 19-9
CBD: Common bile duct
CEA: Carcinoembryonic antigen
CT: Computed tomography
ERCP: Endoscopic retrograde cholangiopancreatography
FDG-PET: ^18^F-fluorodeoxyglucose positron emission tomography
LCNEC: Large-cell neuroendocrine carcinoma
NET: Neuroendocrine tumor
SCNEC: Small-cell neuroendocrine carcinoma
SUVmax: Maximum standardized uptake value
